# Population impact of malaria control interventions in the health district of Kati, Mali

**DOI:** 10.1371/journal.pone.0289451

**Published:** 2024-12-31

**Authors:** Abdoulaye Katile, Issaka Sagara, Mady Cissoko, Cédric Stéphane Bationo, Mathias Dolo, Pascal Dembélé, Bourama Kamate, Ismaila Simaga, Mahamadou Soumana Sissoko, Jordi Landier, Jean Gaudart

**Affiliations:** 1 INSERM, IRD, SESSTIM, ISSPAM, UMR1252, Aix Marseille Univ, Marseille, France; 2 Malaria Research and Training Center (MRTC), FMOS-FAPH, Mali-NIAID-ICER, Université des Sciences, des Techniques et des Technologies de Bamako, Bamako, Mali; 3 National Malaria Control Program, Bamako, Mali; 4 Centre de Santé de Référence du District Sanitaire de Kati, Région de Koulikoro, Mali; 5 APHM, INSERM, IRD, SESSTIM, ISSPAM, UMR1252, Hop Timone, BioSTIC, Biostatistics & ICT, Aix Marseille Univ, Marseille, France; UG NMIMR: University of Ghana Noguchi Memorial Institute for Medical Research, GHANA

## Abstract

**Background:**

WHO and its partners have adopted alternative control interventions since the failure to eradicate malaria worldwide in the 1960s and 1970s. The aim of these interventions has been to redesign the control interventions to make them more effective and more efficient. The purpose of this study is to assess the population impact of control interventions implemented at the community health area level.

**Methods:**

The analysis used data from the health information system on malaria cases and interventions (distribution of long-lasting insecticide-treated nets (LLINs), seasonal malaria chemoprevention (SMC), access to rapid diagnostic tests (RDT), intermittent preventive treatment for pregnant women (IPTp)) collected in the Kati health district from 2017 to 2020. And the contextual parameters (temperature, normal difference vegetation index (NDVI) and rainfall) were obtained by remote sensing. A generalized additive model was used to assess the impact of malaria control interventions on malaria cases as a function of meteorological factors.

**Results:**

The incidence of malaria varies from year to year and from health area to health area, as do meteorological factors in the study area. The distribution of long-lasting insecticide-treated nets, chemoprevention of seasonal malaria in children and access to rapid diagnostic tests for malaria were found to have a significant impact on the incidence of malaria in the population. Seasonal malaria chemoprevention was effective in reducing the incidence of malaria, while distribution of long-lasting insecticide-treated nets and access to rapid diagnostic tests increased with the number of malaria cases, reflecting efforts to distribute and use bed nets and to diagnose malaria cases among the population in the study area.

**Conclusion:**

The study showed the impact of SMC on reducing malaria cases in the population and the significant efforts in LLIN distribution and malaria case diagnosis. To further reduce the burden of malaria, sustained efforts and new interventions are needed, including improving access to rapid diagnosis and treatment in communities by developing community health workers and locally tailored mass drug administration.

## Background

Following the failure to eradicate malaria worldwide in the 1960s and 1970s, WHO and its partners adopted a new set of control interventions, which were updated on a regular basis. The aim of these interventions was to redesign the various control interventions to make them more effective and efficient. These new control interventions focused mainly on controlling morbidity and reducing or even stopping mortality, especially in malaria-endemic areas. To make the interventions more precise, the WHO recommended that they be adapted to the socio-economic and epidemiological factors of different regions of the world. These control interventions were mainly: access to diagnosis and prompt treatment of malaria cases, the use of bed nets and vector control, and preventive measures in high-risk groups [1].

This global control program has had a significant impact on the incidence and prevalence of malaria in the world over the past 20 years. These good results have been the result of good coordination and sustained efforts by WHO and its partners through various targets and actions, including the Roll Back Malaria program launched in 1999, the targets for reducing malaria morbidity and mortality by 2030 [2] and the strengthening and development of new control interventions. These actions, reinforced by initiatives such as the Global Fund to Fight AIDS, Tuberculosis and Malaria, the commitment of African leaders through the Abuja Declaration [3] and the US President’s Initiative to Fight Malaria [4], have made it possible to reduce malaria mortality by 60% in the world and by 44% in Africa. Even more impressive, according to the WHO 2020 report, 21 countries in the world have successfully eliminated malaria [5].

Despite these good results, elimination has not always been achieved, and malaria-related morbidity and disease remain high in endemic areas. Indeed, malaria remains a public health problem and a serious socioeconomic threat in most parts of the world. For example, the number of malaria cases has continued to increase since 2015, with 90% of malaria cases in 2016, 92% in 2017, 93% in 2018, 94% in 2019 and 95% in 2020 [5–9]. And in 2020, 94% of deaths recorded worldwide will occur in the African region. Children under 5 years of age are more affected, with a mortality rate of 77% [5, 9]. In the same year, Mali recorded 843,961 severe cases with 1708 deaths [10]. Furthermore, the funding needed to control and eliminate the disease worldwide has increased significantly over the years, reaching US$1.3 billion in 2017, US$2.3 billion in 2018 and US$3 billion in 2019 [5]. In Mali, the main funding for malaria control comes from the Global Fund and the US President’s Malaria Initiative, representing 36% and 43% of malaria control investment respectively [11].

According to the 2018–2022 Strategic Plan for Malaria Control, malaria control interventions in Mali are essentially based on prevention and management of the disease. This revised plan, extended to 2024, aimed to ensure universal and equitable access to control interventions for the entire population, and provides for the adaptation of control interventions to the new stratification based on the incidence rate. Prevention tools include the use of long-lasting insecticide-treated nets (LLINs) distributed to the general population, intermittent preventive treatment (IPTp) for pregnant women, seasonal malaria chemoprevention (SMC) for children under five years of age, and indoor residual spraying (IRS). The management of malaria-related disease focuses on the systematic confirmation of all suspected cases of malaria by rapid diagnostic tests (RDTs) and early treatment of confirmed cases with artemisinin-based combination therapy (ACT), the control of epidemics and malaria-related emergencies, and the monitoring and evaluation of cases. In addition to these main axes, communication, social mobilization and operational research on new control interventions, drug and vaccine trials are also included [12].

These interventions continue to produce encouraging results, thanks to the commitment of national and international health and political authorities. In most cases, these results vary from year to year and from malaria-endemic area to malaria-endemic area around the world.

In Africa, the number of households using insecticide-treated nets (ITNs) increased significantly between 2000 to 2021. In 2020, 65% of households in sub-Saharan Africa had at least one ITN [9]. In Mali, this rate is 75% in 2018 and 91% in 2021, according to the 2018 Demographic and Health Survey Report [13] and the Malaria Indicators Survey in Mali [14]. Chemoprevention, which aims to prevent and reduce malaria disease and its consequences in the most vulnerable groups by using a combination of antimalarial drugs. In 2019, 49% of pregnant women in Africa received at least two doses of IPTp during their pregnancy and 34% received at least three doses. This 3 dose rate increased slightly from the 2018 rate of 31% [5]; but decreased slightly to 32% in 2020. A similar trend was observed in Mali, where the proportion of pregnant women receiving the three doses of IPTp increased between 2017 and 2018, i.e. 40% and 45% respectively and it decreased, from 45% in 2018 and 35% in 2021 [13, 14]. Seasonal malaria chemoprevention is clearly making progress in the Sahelian region of Africa and has a significant impact on the incidence of malaria in children, and especially on the lethality rate. Several studies conducted in this region have demonstrated its efficacy [15–18]. On the other hand, poor compliance can have a negative impact on the efficacy of this control intervention, and adverse drug reactions such as vomiting, abdominal pain, diarrhea, headache, fever and itching can lead to poor compliance [19, 20]. In 2019, 22 million African children have treated with the SMC and 34 million in 2020 [5, 9]. In Mali, 4 million children were targeted to receive SMC in 2018, and 4.2 million children were treated, representing a coverage rate of 106% [13].

Access to care is a very important factor in the management and prevention of malaria. Not only does it allow diagnosis and early treatment, but it also provides feedback on epidemiological information. However, access to healthcare remains poor in many parts of Africa, particularly in rural areas. This lack of access is mainly due to the absence of local health structures, poor access to health structures due to poor road networks (especially during the rainy season, a period of high malaria transmission, when heavy rains worsen the situation), and the cost of diagnosis and treatment. In 2019, 81% of children under 5 with fever in Africa had sought care, and of these, only 42% had received artemisinin-based combination therapy (ACT). However, in 2020, we saw a decrease in these percentages, with 76% of febrile children seeking care and, only 29% of them had been treated with an ACT [5]. In Mali, according to the Malaria Indicators Survey Report 2021, 60% of febrile children under 5 had sought care and only 19% of these had received ACTs as antimalarial treatment [14].

The High Burden High Impact (HBHI) principle is to focus on local data and information collected in the field [5]. Mali is one of the 10 sub-Saharan African countries that meet the HBHI criteria. It is important to understand and have information at local level on the impact of this control effort in order to better tailor control interventions in a local context. It is in this context that we initiated this work at the level of community health areas, with the aim of assessing the impact of the control measures implemented on the population.

## Methods

### Study site

**Fig 1:**
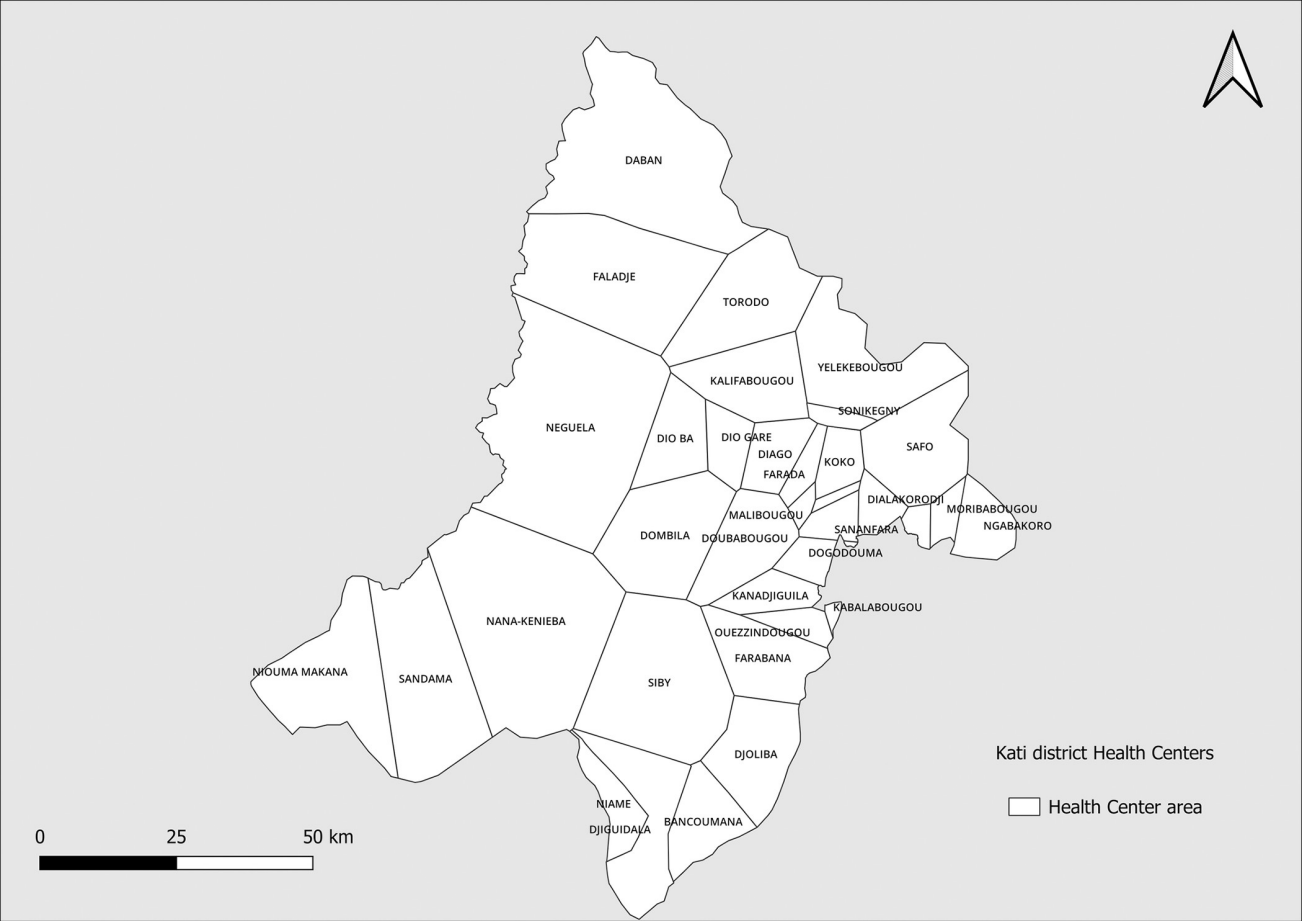
Map of study site: Kati health district with its 35 functional health areas. **Source:** MRTC GIS, **Authors**: Abdoulaye Katile, **Edition**: February 2024.

The study was conducted at the peripheral level according to the Malian health pyramid, in the 35 functional health areas of the 37 health areas of the 23 communes of the Kati health district (Fig 1). In 2019, population of Kati was estimated at 695,921, including 135,429 children under 5, with a density of 66 inhabitants/km^2^, and a growth rate of 3.6%. The district has an area of 9636 km^2^. The Kati health district is located in a Sudano-Sahelian zone where malaria transmission is seasonal and moderate [21]. Annual rainfall averages 1000 mm and occurs between June and September with an average minimum temperature of 20°C and maximum of 35°C. It is an area that is crossed by seasonal streams with few permanent streams. The economic activity is mainly based on the market gardening sector.

### Data collection and source

We analyzed data from the health information system on malaria, and data from remote sensing on meteorological and environmental variables. The study period was at the weekly temporal scale and at the spatial scale of health areas, from January 2017 to December 2020. The following data were extracted from the National Health Information System database (DHIS2: District Health Information System, version 2): number of confirmed malaria cases, number of LLINs distributed during net distribution to children under five years of age and pregnant women, number of children under five years of age treated during the SMC campaign, number of pregnant women treated with IPTp, and total number of RDTs used for malaria case confirmation. The number of people who received LLINs during the campaign was provided by the NMCP activity reports. Meteorological parameters (temperature and rainfall) and Normalized Differentiation Vegetation Index (NDVI) data were obtained from the ERA-5 database of the European Center for Medium-Range Weather Forecasts (ECMRWF) [22]. Monthly data from DHIS2 were aggregated to annual malaria data. The delimitation of each health area was obtained from the geographic coordinates of each health center using the Voronoi polygon method.

### Statistical analysis

A descriptive analysis of the annual malaria incidence rate, control intervention data (LLIN, SMC, IPTp and RDT coverage rates) and meteorological parameters was conducted.

To investigate the association between malaria cases and control interventions, adjusting for meteorological factors, we used the generalized additive model (GAM). This model, with a logarithmic transformation of the population in the offset, allowed us to estimate the standardized incidence ratio. A quasi-Poisson distribution was used to account for overdispersion. Spatial autocorrelation was accounted for by a Gaussian process, using the geographical coordinates of each health center. Spline functions were used to explore the potentially non-linear relationships of factors with malaria. To account for the temporal autocorrelation of malaria incidence, a first-order autoregressive correlation was introduced into the model.

The following model was used:

*Log(Cases)* = *log(pop)* + *f_1_(P)* + *f_2_(longitude, latitude)* + *f_3_(SMC)* + *f_4_(RDT)* + *f_5_(IPTp)* + *f_6_(MILD)* + *f_7_(Rainfall, mean temperature)* + *ε*

Where *pop* is the population during the study period; *P* the study period; *f* the spline functions; ε the residuals.

### Software

The various statistical analyzes were performed using R software version 4.0.5.4 (R Development Core Team, R Foundation for Statistical Computing, Vienna, Austria). The packages used were {mgcv} and {sf}. Maps were produced using QGIS, version 3.20.0 (Open-Source Geospatial Foundation Project, Beaverton, OR, USA). Microsoft Office Paint application was used for image processing.

#### Ethics approval and consent to participate

Permission to use the data analyzed in this study was requested and obtained from the National Malaria Control Program: #00000269/MSDS-SG/PNLP.

## Results

### Descriptive analysis

**Fig 2:**
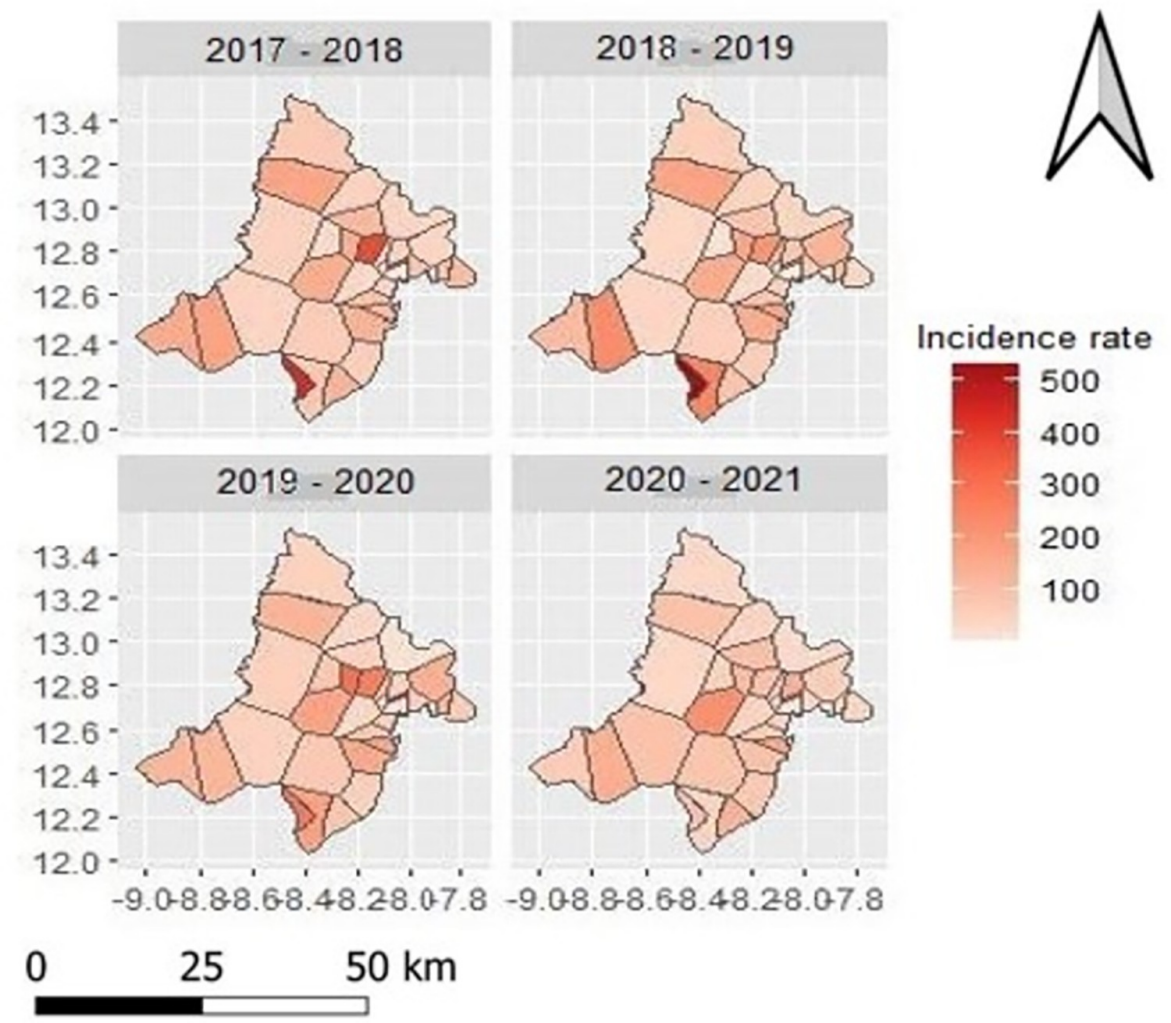
Map of intervention strategies [the distribution rate of LLIN (Long-Lasting Insecticidal Net), the rate of children receiving SMC (Seasonal Malaria Chemoprevention), the number of RDT (Rapid Diagnostic Test) used, the rate of pregnant women receiving IPTp (Intermittent Preventive Treatment of Malaria for Pregnant Women)] per health area. **Source**: MRTC GIS, **Authors**: Abdoulaye Katile, **Edition**: June 2023.

The incidence rate of malaria had decreased from 2017 to 2020 with a maximum of 527 cases per 1,000 person-years in 2018. A significant decrease was observed between 2019 and 2020, from 439 to 270 cases per 1,000 person-years respectively. There is no great variability in mean temperature, vegetation (NDVI) and annual rainfall during the study period (S1 Fig; Table 1). All the control interventions had a coverage rate of over 100% in the years 2020–2021 (Fig 2, Table 1). The average LLIN distribution rate and the average IPTp coverage rate increased over the study period and reached a rate of more than 100% in 2020–2021. However, the average RDT testing rate among suspected cases gradually decreased from the beginning to the end of the study period. At the beginning of the study, and one year after its introduction into the general population (in 2016), the average coverage rate of SMC in the study area increased to 100%. After the first year, this rate gradually decreased to reach a rate of less than 100% in 2020 at the end of the study (Fig 2, Table 1).

**Table 1:** Evolution of the control interventions, the meteorological factors, and the incidence rate of malaria from 2017 to 2021.

	2017–2018				2018–2019				2019–2020				2020–2021			
Variables	Min	Median	Mean	Max	Min	Median	Mean	Max	Min	Median	Mean	Max	Min	Median	Mean	Max
**LLIN**	9.51	26.25	27.32	56.96	4.66	26.54	26.66	46.15	13.94	29.28	30.97	60.65	2.39	36.83	39.41	138.99
**SMC**	59.72	81.40	87.52	159.90	90.76	107.22	114.98	196.58	92.88	103.89	113.26	208.39	70.55	96.36	99.32	141.75
**IPTp**	8.45	34.03	39.08	80.70	6.93	44.25	43.07	105.12	8.06	43.52	44.38	115.88	12.54	42.22	45.98	113.04
**RDT**	4.6	120.8	123.5	235.9	0.46	135.26	123.57	191.49	5.12	116.81	109.87	180.86	2.02	115.76	106.10	194.32
**INCIDENCE RATE**	3.32	67.76	103.21	429.05	1.44	75.81	103.64	527.01	11.06	72.26	103.09	439.19	6.88	60.39	76.13	269.84
**TEMPERATURE (** ^**0**^ **C)**	27	28	28	29	27	28	28	29	28	28	28	29	28	29	29	29
**RAIN FALL (mm)**	745	886	869	959	852	1021	1013	1138	854	997	992	1123	940	1162	1153	1277
**NDVI**	0.46	0.60	0.60	0.67	0.43	0.60	0.59	0.65	0.44	0.61	0.60	0.66	0.42	0.62	0.61	0.66

**Source:** DHIS2; ECMRWF

**LLIN =** Long-Lasting Insecticidal Net coverage rate (%), **SMC** = Seasonal Malaria Chemoprevention coverage rate (%), **IPTp** = Intermittent Preventive Treatment of Malaria for Pregnant Women coverage rate (%), **RDT** = Rapid Diagnostic Test coverage rate (%), **NDVI** = Normalized Difference Vegetation Index, vegetation coverage

### Multivariate analysis

**Fig 3:**
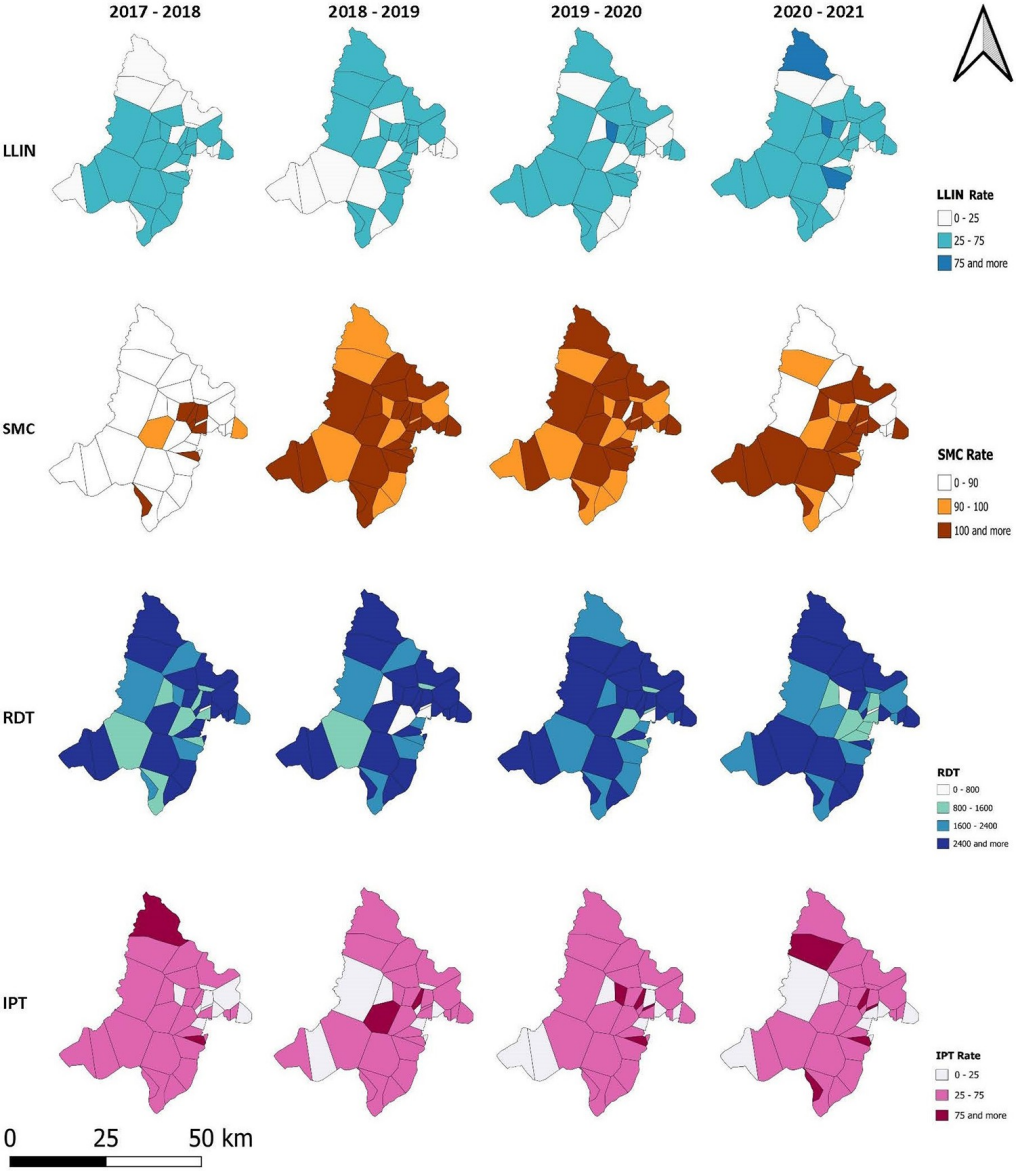
Relationship between malaria incidence (black curve with 95% confidence interval, grey area) and malaria control interventions. **(a)** Seasonal malaria chemoprevention (SMC), **(b)** insecticide treated mosquito nets (MILD) and **(c)** rapid diagnostic test (RDT).

The generalized additive model (GAM) was used to assess the impact of control interventions on the malaria incidence. It found a significant negative linear relationship with the SMC coverage rate, indicating a significant reduction effect on malaria incidence (p = 0.046 Fig 3A). A significant non-linear relationship was found with the LLIN coverage rate (p = 0.002) and the number of RDTs used for malaria case confirmation (p = 0.019). Overall, there is a slight decrease in the incidence rate with increasing LLIN coverage rate. However, the incidence rate tends to increase with the number of RDTs performed (Fig 3B and 3C). The explained deviance was 84%.

## Discussion

**Fig 4:**
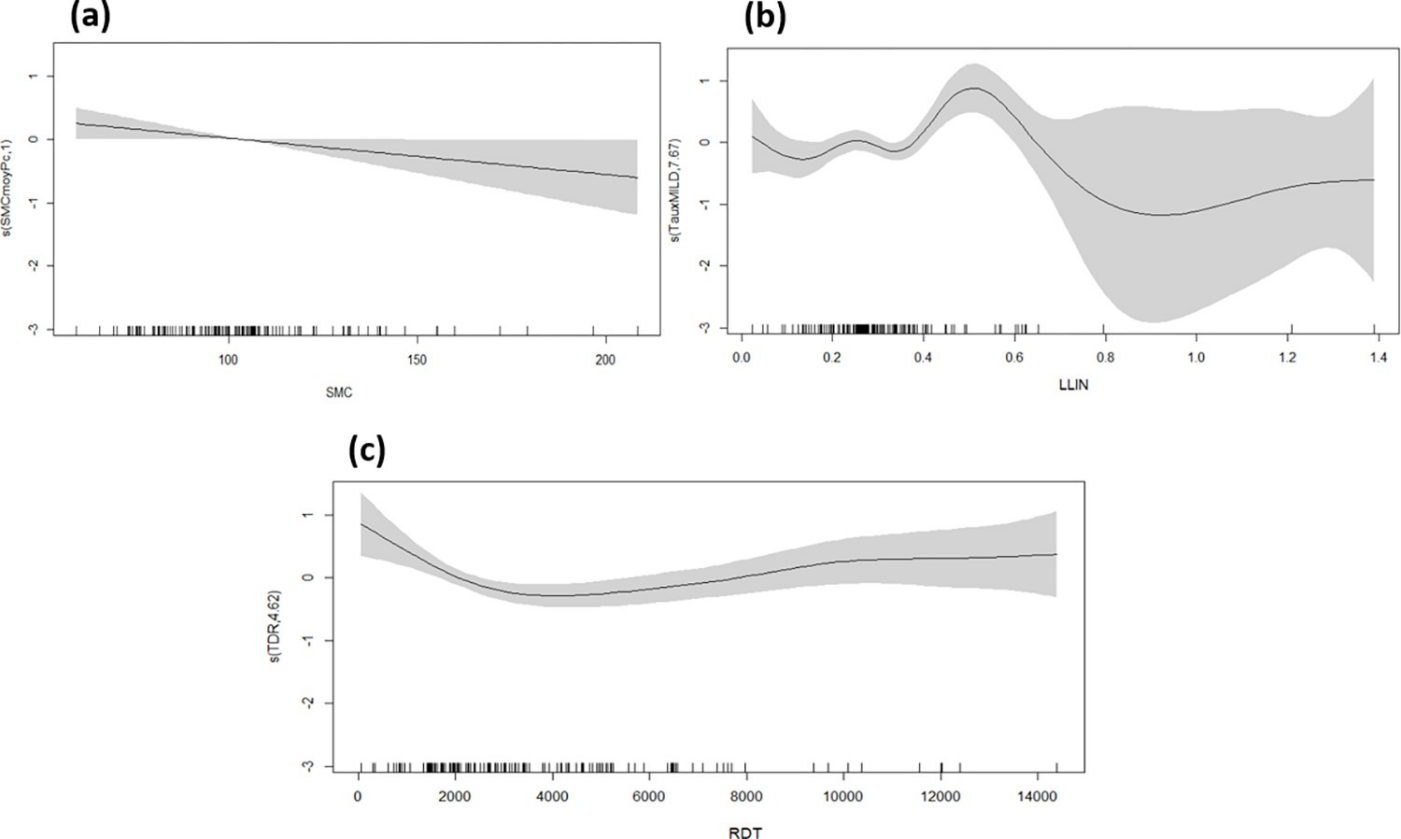
Map of incidence rate of malaria in the study area. **Source**: MRTC GIS, **Authors**: Abdoulaye Katile, **Edition**: June 2023.

The aim of this study was to assess the impact of malaria control interventions on the malaria incidence within the general population, at the level of community health areas in the Kati health district (Fig 1). The descriptive analysis showed that there was a variation in the incidence rate of malaria from one health area to the other in the study area (Fig 4). A significant decrease of malaria incidence rate observed between 2019 and 2020 could be the result of the COVID-19 epidemic, which has an impact not only on the health system but also on social behaviors and activities. Meteorological (rainfall, temperature) and environmental (NDVI) factors did not change significantly over the study period (Table 1; S1 Fig).

We found a significant negative linear relationship between malaria incidence rate and SMC coverage, explaining its effect in reducing malaria cases in the population, even though SMC is targeted at children under 5 years of age. Our result corroborates the findings of several studies demonstrating the positive impact of this intervention tool against malaria in endemic areas. In 2018, Druetz et al, found a protective effect of SMC in a routine national malaria control program in Mali [23]. Furthermore, in a study of routine malaria case data, Sacko et al. observed a decrease in malaria incidence following SMC campaigns in five health districts in Mali with different epidemiological settings of malaria transmission [24]. In Senegal, Ndiaye et al had shown its effectiveness in preventing malaria episodes and reducing parasitemia [25]. In a study conducted in West and Central Africa, Milligan et al. demonstrated the efficacy of SMC in preventing malaria mortality and morbidity [26]. As the age group of SMC is under 5 to 10 years old, they are considered to be the main contributors to the parasite reservoir and contribute to the perpetuating malaria transmission [27, 28]. Treating these children not only protects them but also protects the surrounding population by reducing the number of circulating parasites. However, extending the age limit of children eligible for SMC from 5 to 10 years may not only protect more children, but also further reduce the size of the parasite reservoir [28, 29]. In the literature, several authors have demonstrated the impact of SMC on reducing parasitemia [30–33]. In a study evaluating SMC as part of a routine program in Burkina, Druetz et al demonstrated a 10% reduction in parasitemia during the peak of the transmission season and a reduction in malaria transmission [34]. In Ghana, Tagbor et al. demonstrated the potential for SMC to have a significant public health impact in a region with a long duration of malaria transmission [35]. In 2017, in a pilot study in Niger, Salissou et al found that SMC significantly reduced the parasite load carriage, the number of episodes and incidence of malaria [36]. A non-linear relationship was found between the distribution of LLINs and the incidence rate of malaria, as well as between the coverage of RDTs and incidence rate of malaria. However, we did not find a positive impact (reducing effect) of LLIN distribution in the prevention of malaria cases, and the same was observed for RDT in the management of cases, although it is hoped that prompt treatment will indirectly reduce the number of secondary cases. The slight reduction in the incidence rate with increasing LLIN coverage seems to represent a threshold effect, around 70% coverage. In Uganda, Jagannathan et al found that despite high use of LLINs, the incidence of malaria still remains high [37]. The similar result was found by Ochomo et al in a study carried out in Kenya [38].

As expected, the incidence rate increases with the production of RDTs, indicating the effectiveness of the strategy of rapid access to diagnosis, and may also be explained by confounding bias by indication. SMC, distribution of LLINs and rapid diagnosis of cases by RDTs are very important pillars in the fight against malaria because of their easy implementation and use in the general population. Their effective use can significantly reduce the incidence of malaria in an endemic area. Walker et al demonstrated that LLINs can reduce malaria transmission in the African population at risk [39]. In central Ethiopia, Taffese et al observed a decrease in the incidence of malaria after an LLIN distribution campaign [40].

In this study, we did not find a significant impact of IPTp on the incidence of malaria in the population in the study area. This result may be explained by IPTp use in pregnant women, a restricted population that contributes little to the parasite circulation. In addition, this result may also be explained by the low coverage of prenatal consultation rates, given that IPTp is given during prenatal consultations.

Our result could help the NMCP in the implementation of its national control strategy plan 2018–2022 revised with extension to 2024, which is essentially based on adapting control interventions to the different epidemiological facies obtained during the new stratification of the transmission area.

One of the limitations of our study was limited access to data from remote areas, at the village level. These data are most often collected by community health workers (CHWs) in peripheral areas of health areas. In DHIS2, from which our data were extracted, the routine data collected by CHWs are generally not complete in most health areas. Community health workers (CHWs) play a role in prevention by providing a detailed explanation of the usefulness of mosquito nets and reinforcing their use. They also play an important role in curative care in these remote areas in the diagnosis and rapid treatment and especially the detection of severe malaria for correct and rapid care.

## Conclusion

This study demonstrated that seasonal malaria chemoprevention (SMC), given to children under 5 years of age, has a reducing effect on malaria cases in the population. However, we have observed that the use of insecticide-treated mosquito nets (LLINs) and the rapid diagnostic tests (RDTs) increases with malaria cases, reflecting the efforts made in the distribution and use of mosquito nets as well as the diagnosis of malaria cases in the population of the study area. Thus, strengthening access to diagnostic testing in remote areas, particularly through community health workers (CHWs), under supervision, would contribute to strengthening the curative management of sick persons. But also, prevention, both by reducing the parasite reservoir and by reinforcing messages about the use of bed nets. In addition, it would make it possible to reinforce the detection of severe malaria for referencing. New control strategies should be based on strengthening local interventions to fight malaria in remote areas. such as community health workers and tailored mass drug administrations.

## Supporting information

S1 Fig The map of meteorological factors: Rainfall, NDVI, temperature means. **Source**: MRTC GIS, **Authors**: Abdoulaye Katile, **Edition**: June 2023.
(TIF)

S1 Dataset (XLSX)

## Abbreviations

WHO: Word health organization
LLIN: Long-lasting insecticide net
RDT: Rapid diagnostic test
ACT: Artemisinin-based combination therapy
IPTp: Intermittent preventive treatment of Malaria for Pregnant Women
SMC: Seasonal malaria chemoprevention
ITN: Insecticide-treated net
DHIS2: District health Information System, version 2
NMCP: National malaria control program
ECMRWF: European center for medium-range weather forecasts
GAM: Generalized additive model
CHW: Community health workers
