# Supplementary figures and images for: Population impact of malaria control interventions in the health district of Kati, Mali
Source: PLoS One. 2024 Dec 31;19(12):e0289451. doi: 10.1371/journal.pone.0289451 (PMC11687661; doi:10.1371/journal.pone.0289451)

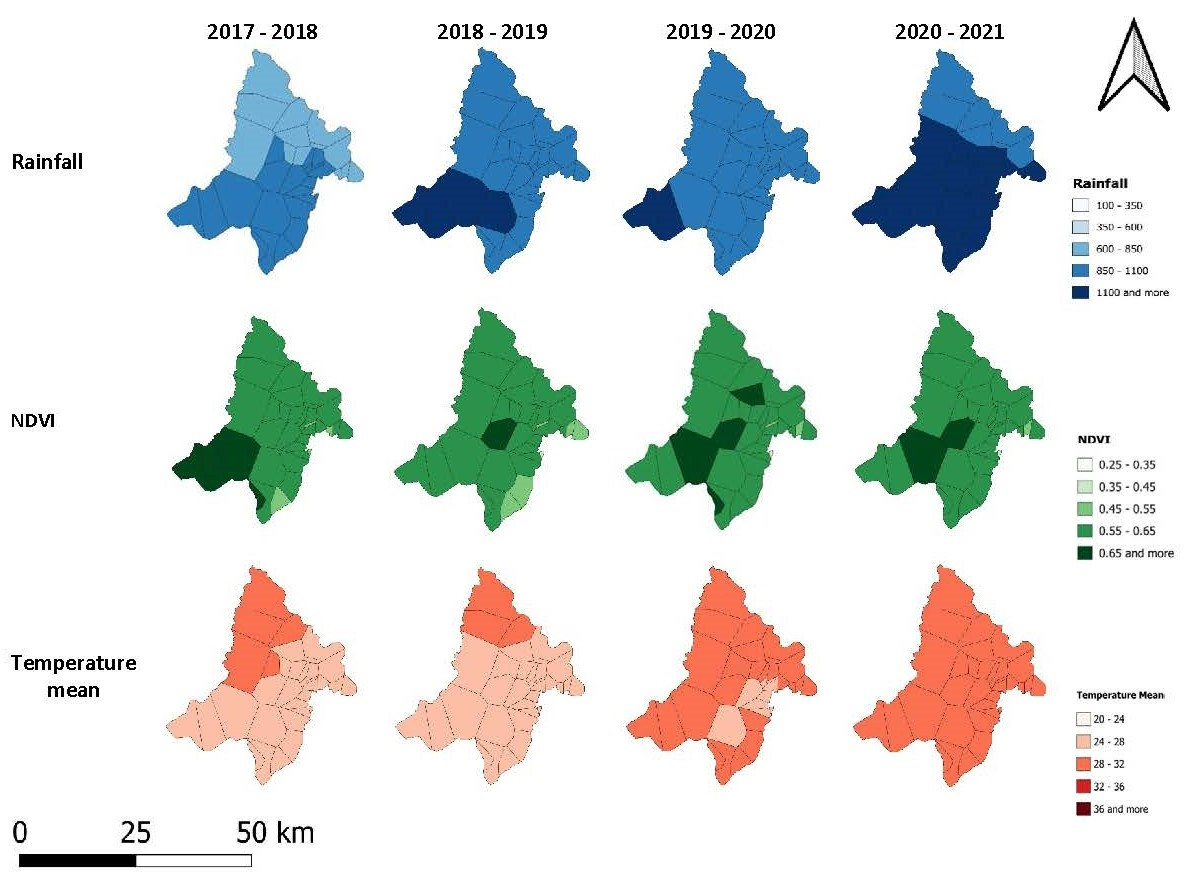

Supplement: S1 Fig — Source: MRTC GIS, Authors: Abdoulaye Katile, Edition: June 2023. (TIF) [file pone.0289451.s001.tif]
